# New insights into hypothalamic neurogenesis disruption after acute and intense stress: implications for microglia and inflammation

**DOI:** 10.3389/fnins.2023.1190418

**Published:** 2023-06-23

**Authors:** María Inmaculada Infantes-López, Andrea Nieto-Quero, Patricia Chaves-Peña, Emma Zambrana-Infantes, Manuel Cifuentes, Javier Márquez, Carmen Pedraza, Margarita Pérez-Martín

**Affiliations:** ^1^Departamento de Biología Celular, Genética y Fisiología, Universidad de Málaga, Málaga, Spain; ^2^Instituto de Investigación Biomédica de Málaga y Plataforma en Nanomedicina–IBIMA Plataforma Bionand, Málaga, Spain; ^3^Departamento de Psicobiología y Metodología de las Ciencias del Comportamiento, Universidad de Málaga, Málaga, Spain; ^4^Departamento de Biología Molecular y Bioquímica, Canceromics Lab, Universidad de Málaga, Málaga, Spain

**Keywords:** neurogenesis, microglia, inflammation, acute stress, hypothalamus, hypothalamic proteomic profile

## Abstract

In recent years, the hypothalamus has emerged as a new neurogenic area, capable of generating new neurons after development. Neurogenesis-dependent neuroplasticity seems to be critical to continuously adapt to internal and environmental changes. Stress is a potent environmental factor that can produce potent and enduring effects on brain structure and function. Acute and chronic stress is known to cause alterations in neurogenesis and microglia in classical adult neurogenic regions such as the hippocampus. The hypothalamus is one of the major brain regions implicated in homeostatic stress and emotional stress systems, but little is known about the effect of stress on the hypothalamus. Here, we studied the impact of acute and intense stress (water immersion and restrain stress, WIRS), which may be considered as an inducer of an animal model of posttraumatic stress disorder, on neurogenesis and neuroinflammation in the hypothalamus of adult male mice, focusing on three nuclei: PVN, VMN and ARC, and also in the periventricular area. Our data revealed that a unique stressor was sufficient to provoke a significant impact on hypothalamic neurogenesis by inducing a reduction in the proliferation and number of immature neurons identified as DCX+ cells. These differences were accompanied by marked microglial activation in the VMN and ARC, together with a concomitant increase in IL-6 levels, indicating that WIRS induced an inflammatory response. To investigate the possible molecular mechanisms responsible for neuroplastic and inflammatory changes, we tried to identify proteomic changes. The data revealed that WIRS induced changes in the hypothalamic proteome, modifying the abundance of three and four proteins after 1 h or 24 h of stress application, respectively. These changes were also accompanied by slight changes in the weight and food intake of the animals. These results are the first to show that even a short-term environmental stimulus such as acute and intense stress can have neuroplastic, inflammatory, functional and metabolic consequences on the adult hypothalamus.

## 1. Introduction

Stress can have an impact on the structure and function of the brain and can result in changes in mood and motivation, among other behaviors (Pizzagalli, 2014). The hypothalamus is considered the center of the limbic system (RajMohan and Mohandas, 2007). Situated at the convergence of many neural pathways, the hypothalamus serves as a regulator of several critical homeostatic processes and mediates emotional responses mainly through the autonomic nervous system (McCorry, 2007). There, many hormonal and autonomic responses take place, which seem to be key for controlling the stress response system (Ulrich-Lai and Herman, 2009; Godoy et al., 2018). In fact, hypothalamic outputs regulate functions that are significantly impaired by stress, such as metabolic, endocrine, behavioral, and sexual functions (Jurkowski et al., 2020). However, the neurobiological changes that take place in the hypothalamus as a consequence of stress are not known in detail.

In recent decades, the hypothalamus has emerged as a new neurogenic area, containing new neurons generated after early development (Zhan et al., 2013; Jurkowski et al., 2020). The presence of neural stem and progenitor cells (NSPCs) in this area suggests the existence of a neurogenic niche with the ability to replicate and populate local neural circuits (Xu et al., 2005; Pérez-Martín et al., 2010; Lee et al., 2012). Although the level of neurogenesis is low compared to those detected under basal physiological conditions in other conventional neurogenic regions, such as the hippocampus, there is a growing interest in clarifying the process of hypothalamic neurogenesis because of the functional implications of this region. Neurogenesis-dependent neuroplasticity is crucial for the hypothalamus to continuously adapt to internal and environmental changes (Pierce and Xu, 2010; Ming and Song, 2011).

The effects of stress on hippocampal neurogenesis are widely known, and it has been described that stress strongly suppresses adult hippocampal neurogenesis (Schoenfeld and Gould, 2012; Lu et al., 2014; Llorens-Martín et al., 2016; Ito et al., 2017; Lee and Giuliani, 2019), as these cellular disturbances are linked to the neuropathology of stress-related disorders (Campbell and Macqueen, 2004; Lucassen et al., 2010; Snyder et al., 2011). Similar to the hippocampus, various factors have emerged as potent modulators of hypothalamic neurogenesis. In fact, hormones such as gonadal hormones (Yoo and Blackshaw, 2018), behavioral and environmental stimuli such as social isolation (Lieberwirth et al., 2012), changes in diet, mainly fat-based diet (McNay et al., 2012), early life stress (Kisliouk et al., 2014), heat stress (Bielefeld et al., 2021), can affect hypothalamic neurogenesis. It is therefore not unreasonable to think that stress may have an impact on hypothalamic neurogenesis. Therefore, as a structure involved in the control of the organism’s homeostasis, its study is of great interest.

On the other hand, in the hippocampus, although not entirely conclusive, it appears that changes in neuroinflammation mediated primarily via microglial cells may play an essential role in regulating neurogenesis (Ekdahl et al., 2003; Monje et al., 2003; Nieto-Quero et al., 2021). However, it is not known whether microglial changes also mediate the effects of stress on hypothalamic neurogenesis. Although there are numerous studies aimed at understanding the neuroinflammatory response in brain regions involved in emotional regulation (hippocampus, prefrontal cortex, etc.), the information available on changes at the level of the hypothalamus is scarce, and it is unknown whether, as in the hippocampus, there is a possible relationship between microglial response to stress and hypothalamic neurogenesis.

Since the study of acute stress-induced effects can provide valuable information on the mechanisms involved in the stress response and pathological responses to stress, we set out to study the neurogenic changes and neuroinflammatory response in the hypothalamus as a consequence of exposure to acute and intense stress, which can act as a model of posttraumatic stress disorder (PTSD), an anxiety disorder associated with biological disturbances, with the hypothalamus being a particularly affected structure (Mironova et al., 2017). We have also addressed whether changes in hypothalamic microglia mediate neurogenic changes. For this purpose, we have used an animal model of acute water immersion and restraint stress (WIRS), whose application has been associated with microglial morphological changes (Sugama et al., 2007), neuroinflammatory conditions and hippocampal neurogenic alterations (Mao et al., 2020), and numerous neuroendocrine and neurotransmitter system impairments that have been associated with numerous psychopathological disorders (Ohta et al., 2017; Yasugaki et al., 2019) such as anxiety-like, depression-like and memory impairment phenotypes (Purwoningsih et al., 2022). In these animals, we have measured neurogenic changes, analysing cell proliferation and immature neurons, inflammatory changes, hypothalamic microglial response and the relationship between both processes in three hypothalamic nuclei key in emotional regulation and stress response. Specifically, we investigated the paraventricular nucleus (PVN) which is directly involved in regulating the hypothalamic–pituitary–adrenal axis (HPA) (Herman et al., 2016), the ventromedial nucleus (VMN), because of its relation to food intake and metabolism (Eghbal-Ahmadi et al., 1997; Tasker and Joëls, 2015), the arcuate nucleus (ARC), which is crucial for the maintenance of energy homeostasis (Yi et al., 2006), and the periventricular region (PE) which is consider a proliferative area of the adult hypothalamus (Lee and Ahima, 2012). To better characterize the response to stress, we studied proteomic changes 1 h and 24 h after stress. Weight and food intake were recorded after exposure to stress to explore metabolic consequences.

## 2. Materials and methods

### 2.1. Protocols for the histological study

All animal procedures were conducted following the European regulations for animal research (General Rules from the European Community Council 2010/63/UE, 90/219/CEE, Regulation (EC) No. 1946/2003) and Spanish National Guidelines for Animal Experimenting (Real Decreto 53/2013), approved by the University of Malaga Ethical Committee (CEUMA 51-2018-A) and corroborated by the Agriculture, Farming, Fishing and Sustainable Development Council (10/06/2019/114).

#### 2.1.1. Animal experimental protocol

For this experiment, a total of 16 male C57BL/6 J mice that were 3 m.o. (Charles River Laboratories, Inc., Wilmington, Massachusetts, United States) were used following standard housing conditions (RT = 22 ± 2°C, HR = 55 ± 5%, 12 h light/dark cycle) with food and water administered *ad libitum.* Animals were caged individually. The experiments were carried out in the morning from 9:00 to 13:00.

##### 2.1.1.1. Experimental groups

**Figure 1:**
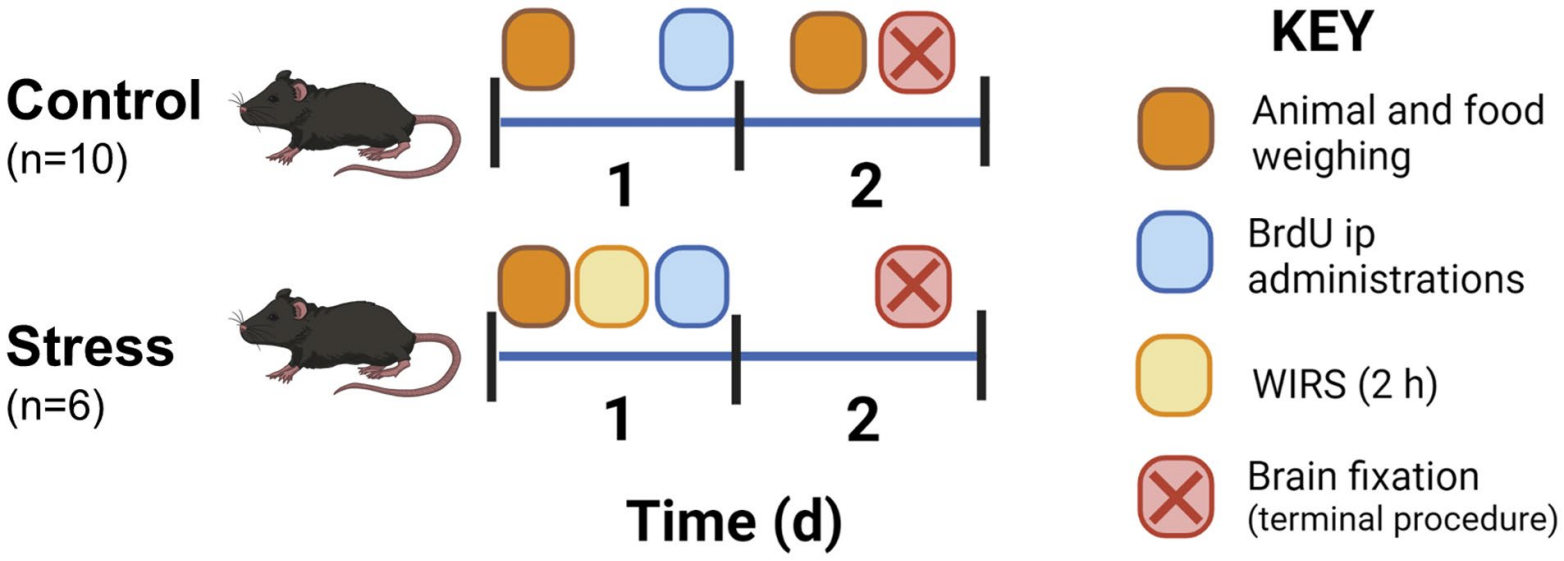
Experimental protocol for the animal procedure including sampling, injections, and experiment termination for histological studies. Animals were handled 1 week prior to the experiment to habituate the animals to researcher contact.

Animals were initially separated into control and stress groups. Here, we used the combined water immersion and restriction stress (WIRS) paradigm. The stress animals (*n* = 6) suffered a unique stress episode for 2 h, whereas the control animals (*n* = 6; except for the hypothalamic microglia characterization study in the control situation where a total of 10 control animals were used) remained home-caged (Figure 1). Prior to the experimental procedures, the animals were handled for 1 week to reduce manipulation stress. Animal weight was measured before and 24 h after stress. To calculate food intake, food was weighed daily before all other procedures from the day before the stress protocol until brain fixation. Food intake was calculated as the net amount of food eaten in 24 h.

##### 2.1.1.2. Stress protocol

Stress was conducted on Day 1 of the experimental design (Figure 1). WIRS consisted of introducing the mouse into a narrow perforated tube where movement was limited yet breathing was enabled (Miyata et al., 2011; Ohgidani et al., 2016). Then, animals were placed vertically in room temperature water that contained the recipient at the level of the chest for 2 h. Animals were then returned to their home cage. Control animals were home-caged for the duration of the stress procedure.

##### 2.1.1.3. 5-bromo-2′-deoxyuridine administration

Following the stress session, the animals received an intraperitoneal injection with a thymidine analog. Overall, three consecutive injections were administered with a 3 h interval between injections. 5-bromo-2′-deoxyuridine (BrdU, Sigma–Aldrich, Madrid, Spain) (50 mg/kg in NaCl 0.9%) was injected on Day 1 into all groups after the stress procedure. This was done for the purpose of identifying cells newly generated after stress.

##### 2.1.1.4. Perfusion

Animals were anaesthetized using 200 mg/kg sodium pentobarbital (ip. injected), and the circulatory system was washed using PBS, pH 7.4. Later, animals were perfused using a 4% PFA solution for fixation. Brains were extracted from the skull and left for postfixation for an additional 24 h at 4°C. They were changed to PBS + 0.02% sodium azide at 4°C.

#### 2.1.2. Histological preparation of the tissue

##### 2.1.2.1. Brain sectioning

Brains were embedded in 4% agar prior to sectioning using a Leica VT1000S vibratome. Whole brains were sliced in coronal 40 μm thick slices and stored in six series. Thus, each section was 240 μm apart from the next section within each series for the histological analysis. Sections were stored in cryoprotectant solution (30% ethylene glycol, 30% sucrose) at −18°C until further use.

##### 2.1.2.2. Immunohistochemical staining

Several histological markers were analysed using a rabbit anti-Iba-1 IgG antibody for microglia (Wako, United States, cat. # 019-19741, 1:500 dilution), a rat anti-BrdU IgG antibody (Accurate Chemicals, Westbury, NY, United States, cat. # OBT0030, 1:1,000 dilution) for new cells and a goat anti-doublecortin IgG (DCX) antibody (Santa Cruz Biotechnology Inc., Dallas TX, United States, cat. # sc8066, 1:200 dilution) for immature neuron identification. The following biotinylated secondary antibodies were used at a 1:1,000 dilution: goat anti-rabbit IgG (Dako, CA, USA, cat. # E0432), rabbit anti-rat IgG (Thermo Fisher Scientific, Rockford, United States, cat. # 31834), and donkey anti-goat IgG (Thermo Fisher Scientific, Rockford, United States, cat. # PA1-28663, donkey).

First, slices were washed in PBS three times for 10 min with agitation at room temperature. For the BrdU protocols, an additional step of antigen retrieval was necessary. It consisted of a 15 min incubation in 2 N HCl at 37°C followed by three quick PBS washes and three additional 10 min washes. Then, endogenous peroxidase was inactivated (10% H_2_O_2_, 10% methanol in PBS) by agitation for 45 min away from light at room temperature. After three 10 min washes in PBS, tissue saturation was followed by a 1 h incubation in PST (2.5% sheep serum, 0.75% Triton-X100 in PBS). Tissue was later incubated in primary antibody solution in PST o/n at 4°C with agitation. The next day, the primary antibody solution was removed, and after three washes in PBS, the secondary antibody was added and incubated for 90 min with agitation at room temperature. After three washes in PBS, an ExtrAvidin-Peroxidase solution (Sigma–Aldrich, cat. # E2886, 1:1,000 in PBS) was incubated for 1 h at room temperature with agitation. Finally, after washing the tissue three times, the brain slices were visualized using 2.5% 3,3-diaminobenzidine (DAB) and 0.1% H_2_O_2_ in PBS for 6.5 min. Later, slices underwent three quick washes and three 10 min washes. For DCX, DAB was supplemented with 0.4% NiCl_2_ to intensify staining. Slices were mounted on a gelatine-coated slide following the rostrocaudal axis. For Iba-1 slides, a contrast staining was performed by staining for 5 s in 0.3% toluidine blue. Slides were dehydrated and mounted using Eukitt mounting media. For the negative control, the primary antibody was omitted.

The immunofluorescence protocol was similar to the previous protocol. The differences are as follows. No endogenous peroxidase inactivation step was needed, and therefore the tissue was directly blocked after antigen retrieval and subsequent washes. The same primary antibody and dilution for BrdU and DCX were used. In this case, they were incubated together in the same solution o/n at 4°C. Secondary antibodies Alexa Fluor 488 donkey anti-rat IgG (Invitrogen, cat. # A21208) and Alexa Fluor 647 donkey anti-rabbit IgG (Invitrogen, cat. # A31573) were used at a 1:1,000 dilution. After secondary antibody incubation and during the second wash, sections were incubated with DAPI (Sigma, Madrid, Spain) (1,50,000 dilution) for 20 min followed by a regular PBS wash for 10 min. Sections were imaged using confocal microscopy (Leica Stellaris 8, ×40 lens).

##### 2.1.2.3. Hypothalamic regions of interest

**Figure 2:**
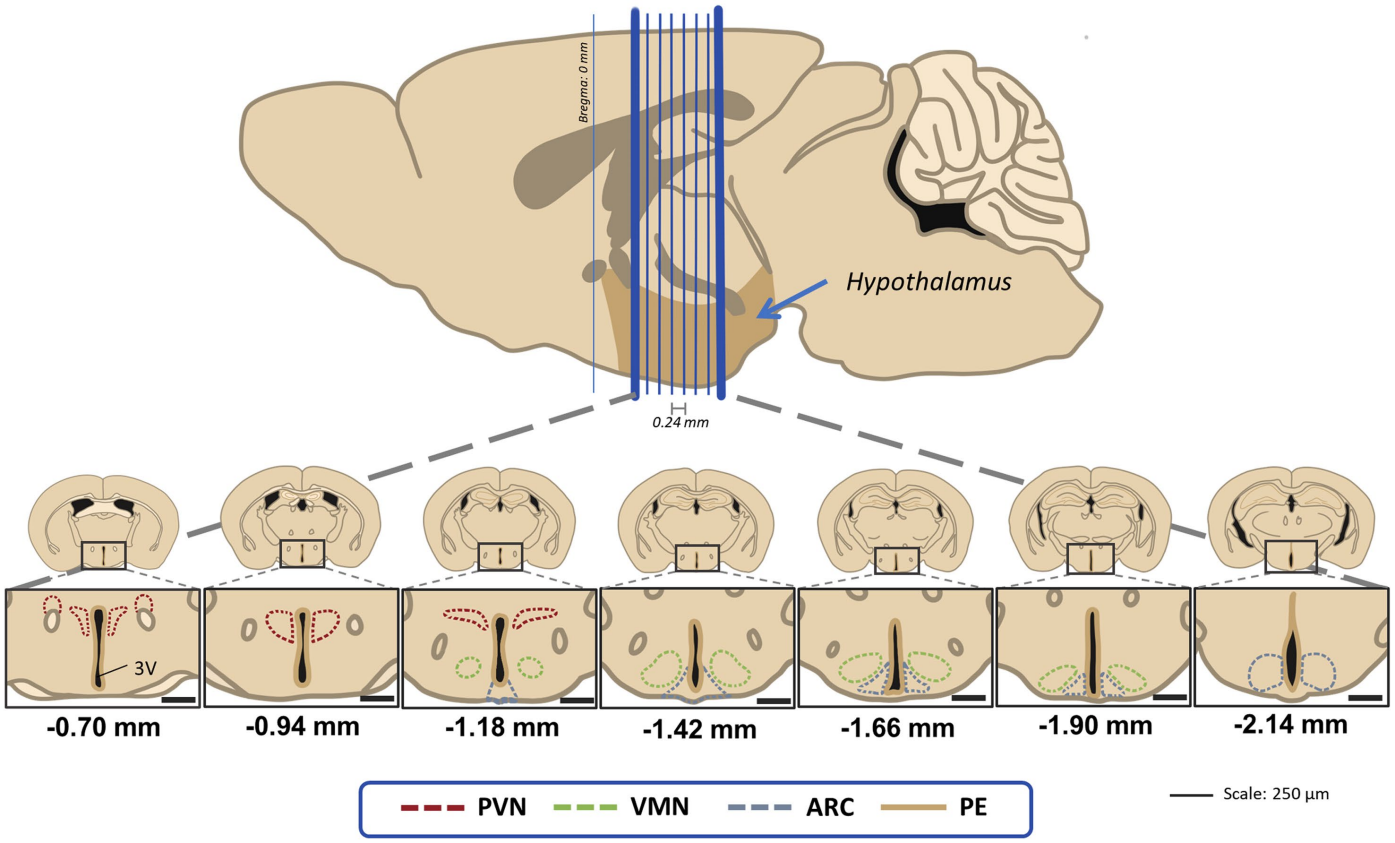
Visual guide for the hypothalamic regions of interest in this study for the 40 μm coronal sections that were 240 μm apart from the next one. Distances are marked using Bregma as reference as stated in the stereotaxic atlas (Paxinos and Franklin, 2004). PVN, paraventricular nucleus; VMN, ventromedial nucleus; ARC, acuate nucleus; PE, periventricular hypothalamic nucleus.

For each animal, up to seven slices were studied to account for three hypothalamic regions: paraventricular nucleus (PVN), ventromedial nucleus (VMN) and arcuate nucleus (ARC) following Paxino’s Atlas reference (Paxinos and Franklin, 2004). Sections covered −0.70 mm to −2.18 mm Bregma in 240 μm intervals from one slice to the next (Figure 2).

#### 2.1.3. Proliferation analysis using BrdU and immature neurons using DCX

For this study, four animals per group were randomly selected (*n* = 8). After administration of the thymidine analog BrdU after stress, proliferation was measured by manually counting cells on a bright field microscope (DMLB microscope (Leica, Wetzlar, Germany, ×40 lens)). The average number of BrdU+ cells per section was determined. DCX+ cells were manually identified and counted using the same microscope setup.

Only one hemisphere was considered for PVN, VMN, ARC and PE BrdU and DCX cell counts. Brain regions were delimited following the stereotaxic atlas as a reference (Paxinos and Franklin, 2004). Due to the majority of BrdU and DCX cells being in the periventricular region (PE), this area was also included in the study.

#### 2.1.4. Microglial study using Iba-1

##### 2.1.4.1. Image scanning

Slides were scanned at ×20 magnification using an Olympus VS120-5S and *VS*-ASW Software (Olympus, Tokyo, Japan). For each slice, a 10 μm range was scanned, and that stack was combined into one single image.

##### 2.1.4.2. Soma morphological analysis

**Figure 3:**
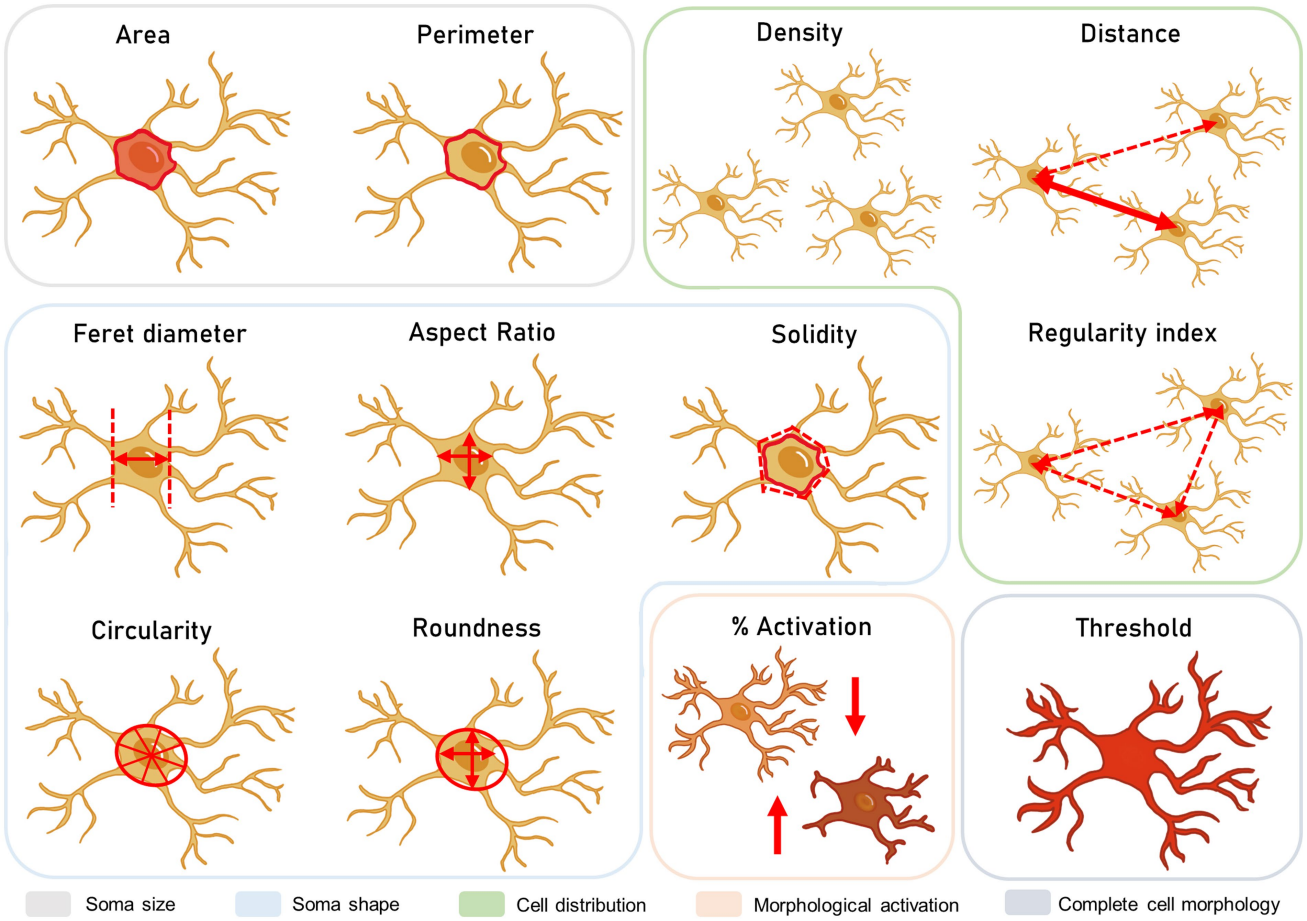
Microglial morphological study. These parameters include the size, shape, and distribution of the cells, as well as the integration of morphology parameters in percentage of activation and total area stained.

To study the morphology and distribution of microglia under basal conditions (in absence of stress) in the three hypothalamic nuclei, a total number of 10 animals was used. For Control-Stress comparison, a number of 6 animals for group was included (*N* = 12). Using ImageJ Fiji (Schneider et al., 2012) analysis tools according to Davis et al. (2017), hypothalamic regions of interest were marked on each image. Thus, the software automatically detected cell somas within 15–200 px^2^ and with a greyscale filter from 0 to 180. Overall, 10 parameters were analysed, including area, perimeter, circularity, Feret diameter, aspect ratio, solidity, roundness, distance (to the closest cell), density and regularity index. The regularity index was calculated by dividing the *distance*’s standard error by the total number of microglia in that area (Figure 3).

##### 2.1.4.3. Morphological activation phenotype clustering

Here, animal numbers and distribution were equal to soma morphological analysis. Cells were individually classified into a high or low activated phenotype based on morphological changes in the soma area and roundness, as previously described (Davis et al., 2017). High morphological activation accounted for a higher soma area and lower roundness, which is related to an activation in cell motility. These cells were considered stress-reactive microglia (Paolicelli et al., 2022). A lower activation phenotype described the opposite phenotype, associated with more static microglia. Cells were classified using K-means clustering for two clusters and a maximum of 20 iterations to find centroids in the clusters in IBM SPSS Statistics 20.

##### 2.1.4.4. Threshold analysis for total microglial staining

For this measurement, a total number of *n* = 6 animals per group was included (*N* = 12). As a quantitative approximation of microglial processes, a threshold analysis was performed (Figure 3). Together with the soma analysis, information on the ramifications could be inferred, somewhat similar to the ramification index in previous studies (Plescher et al., 2018). In this case, scanned images were binarized and filtered so that total Iba-1 stain was included in the measurement. Then, greyscale intensity for each value of the scale (0–255) was measured for each image so that a slope could be obtained for total Iba1 staining in ImageJ Fiji. Intensity values were normalized by image total area according to the analysed region. By comparing cell soma areas and total thresholds, changes in microglial ramifications could be interpreted.

### 2.2 Protocols for the molecular study

A second block of experiments was proposed to study the effect of acute stress on molecular parameters that may explain or mediate hypothalamic cellular changes. Thus, a more in-depth analysis in terms of the temporality of changes was performed.

#### 2.2.1 Animal experimental protocol

**Figure 4:**
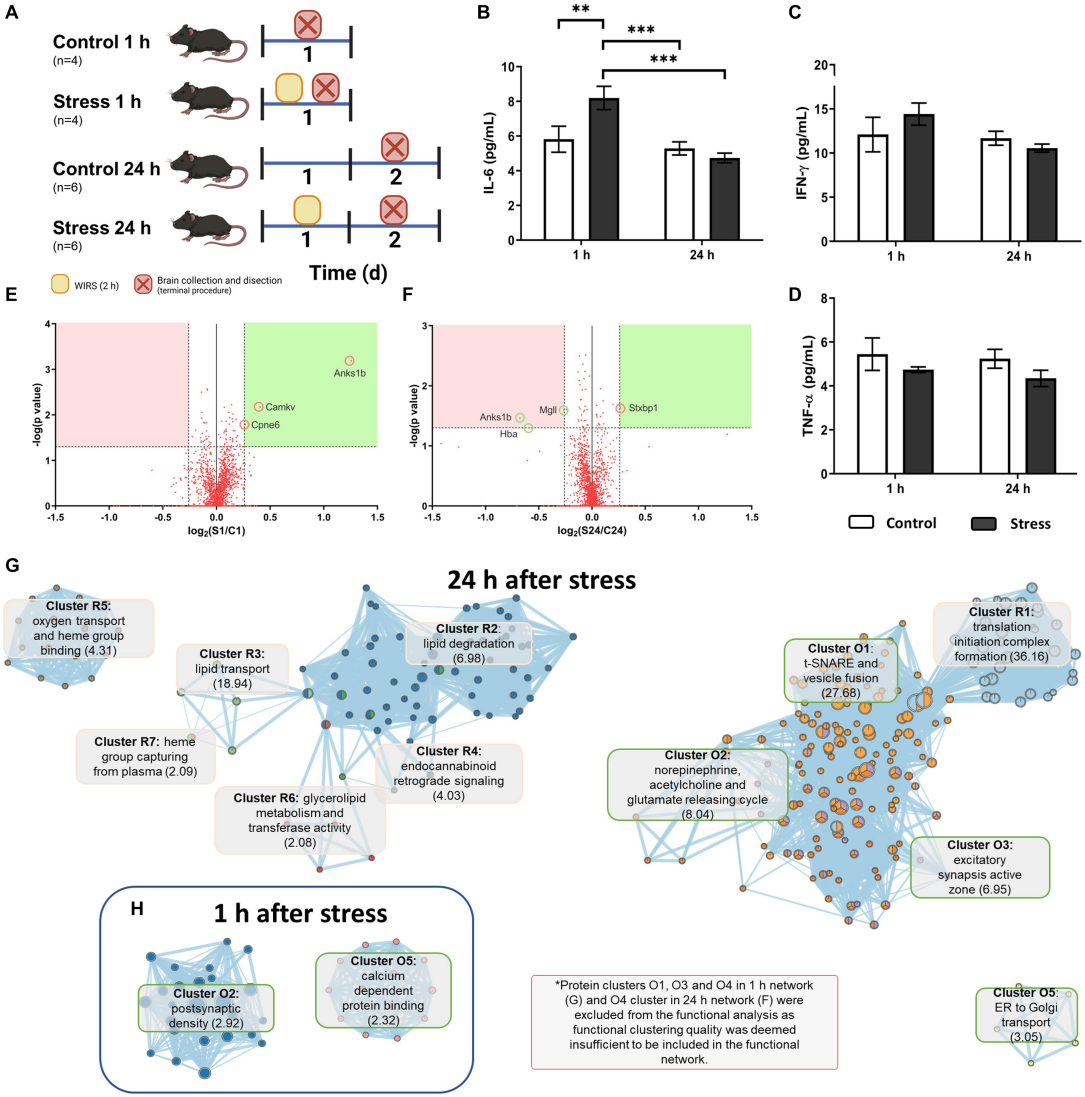
Molecular analysis of acutely stressed mice 1 h and 24 h after stress. **(A)** Experimental mouse model and animal count. **(B–D)** Cytokine concentrations in brain tissue. Two-way ANOVA and LSD Fisher *post hoc* analysis (**p < 0.005; ***p < 0.0005). **(E,F)** Volcano plots for proteomic findings 1 h **(E)** and 24 h **(F)** after stress according to the indicated selection criteria. **(G,H)** Functional network for affected proteins 1 h **(H)** and 24 h **(G)** after stress obtained with the EnrichmentMap plug-in from Cytoscape. Clusters starting in R and in pink represent repression in stressed animals, and those starting in O and in green represent overexpression in stressed animals. The main GO term score in clusterization by DAVID is indicated in brackets.

A different cohort of animals was used for this study (*n* = 4–6 per group, *N* = 20), following a stress procedure similar to the histological study but with different times of perfusion (1 h or 24 h after stress) (Figure 4). Animals were euthanized using the same dose of sodium pentobarbital as described above. Brains were perfused with PBS, dissected, collected and stored at −80°C for later processing.

#### 2.2.2. Cytokine level measurements

Inflammatory cytokines (IL-6, TNF-α, and IFN-γ) were measured in the left hypothalamus. Brain tissue was homogenized in lysis buffer (125 mM NaCl, 1 mM EGTA, 1 mM EDTA, 20 mM HEPES pH 7) and then centrifuged at 14,000 × g for 15 min at 4°C for supernatant recovery. The lysate was brought to a concentration of 7 μg/mL total protein. Next, a ProcartaPlex^™^ Multiplex Immunoassay (Invitrogen, Thermo Fisher Scientific, Massachusetts, United States) was conducted following the manufacturer’s instructions. Reads were performed using a Luminex^™^ instrument (Bio-Plex^™^ 200 System, Bio-Rad, California, United States). Lower limits of quantification were 0.470 pg./mL for IL-6, 0.313 pg./mL for TNF-α and 18.768 pg./mL for IFN-γ. Statistical analysis was performed using the software StatSoft Statistica 8 and one-way ANOVA (*p* < 0.05). A *post hoc* LSD Fisher analysis was performed to determine differences among groups.

#### 2.2.3. Hypothalamic proteomic profiling using mass spectrometry

Right hypothalami from four animals per group were processed for protein extraction using TEAB-SDS buffer and centrifuged at 16,000 × g for 10 min at 4°C to recover the supernatant. The protein concentration was calculated by the BCA method and adjusted to 1 μg/mL. Protein extracts were readied using an isobaric tandem mass tag procedure (TMT 6 plex, Thermo Fisher Scientific). Samples were fractioned using a reversed-phase column in a high pH and a Thermo Scientific Pierce High pH Reversed-Phase Peptide Fractionation Kit, obtaining 8 fractions per sample for better resolution.

Then, a hybrid Quadrupole-Orbitrap Mass Spectrometer (Q-Exactive HF-X, Thermo Fisher Scientific) coupled with an EASY-Spray^™^ (Thermo Fisher Scientific) ionization source and Easy-nLC 1200 UHPLC system (Thermo Fisher Scientific) was used for mass identification and relative quantification of proteins with a shotgun procedure. The SwissProt database for *Mus musculus* (ver 2017.10.25) was used to identify the 4,529 proteins found. Identification criteria also included those matched to a “Master” protein, an FDR < 0.01 and at least 2 unique peptides found for the protein, which reduced the total number of proteins to 1,850. For data analysis, the level of each protein was normalized to the total peptide concentration and later normalized to all the rest of the proteins. Ratios for experimental and control samples were calculated using the *protein abundance-based* method. Selection criteria for proteins included *p* < 0.05 and FC (fold change) > 1.200 for overexpression and FC < 0.833 for repression after stress (Dozio and Sanchez, 2018).

#### 2.2.4. Protein clustering and functional analysis

Proteins meeting the criteria outlined above were analysed by STRING 11.0 (Szklarczyk et al., 2019), and a first shell was added with 100 interactors for each category (overexpression/repression). This enriched list of proteins was analysed in the MCODE (Bader and Hogue, 2003) plugin for Cytoscape 3.8.2 (Shannon et al., 2003) to find highly connected nodes in the networks. For this, proteins belonging to each cluster as well as cluster information were obtained (loops and fluff not included, haircut performed, node density cut-off = 0.2 and degree cut-off = 2). Each cluster protein list was submitted to the DAVID Database (Dennis et al., 2003) so that a functional analysis could be performed. GO terms associated with genes were analysed in the EnrichmentMap (Merico et al., 2010) plugin in Cytoscape 3.8.2 (overlap threshold = 0.6, *p* = 0.001 and FDR *q*-value < 0.05 for a restrictive interpretation of data) to obtain functional networks for protein clusters.

### 2.3. General statistics

Data compilation and mean/SEM calculation were performed using Excel 2016. T-student tests were used for two-group comparisons (control vs. stress), whereas one-way ANOVA tests were used to compare the three regions in the control group. Due to inter-individual variability in feed intake and body weight in animals, for food intake and body weight, ANCOVA was performed to determine whether weight differences were due to the treatment and not animal variability prior to the stressor. Data collected prior to stress were treated as a covariate, and differences between the control and stress groups after stress were analysed. These analyses were performed using StatSoft STATISTICA 8. For all tests, a 95% confidence interval was set. Outlier values were removed from the analysis using IBM SPSS Statistics 20.

Additionally, a principal component analysis (PCA) with varimax rotation was performed to make associations with all different datasets to group them into factors and study their relationship (Balaban et al., 2014). Input data variables included soma area, density, microglial activation, DCX+ and BrdU+ cell counts and final body weight. The correlation matrix of the entire sample of animals (*n* = 12) was used for the analysis and tested for sampling adequacy by Bartlett sphericity and the statistical adequation of the samples. The Kaiser–Meyer–Olkin sample adequation index (KMO) was studied and considered for values >0.5 (Balaban et al., 2014). The resulting factors with eigenvalues>1 were selected. “Factor loading” (i.e., the contribution of each variable to a factor was considered significant if it was >0.60). Finally, considering that the component or factor scores represented the relative contribution or weight of each loading pattern for each case, a student *t* test was used to determine whether differences existed between the groups in each loading pattern. This was conducted in IBM SPSS Statistics 30.

Data were considered statistically significant at *p* ≤ 0.05.

## 3. Results

### 3.1. Stressed animals had reduced appetites and body weights

**Figure 5:**
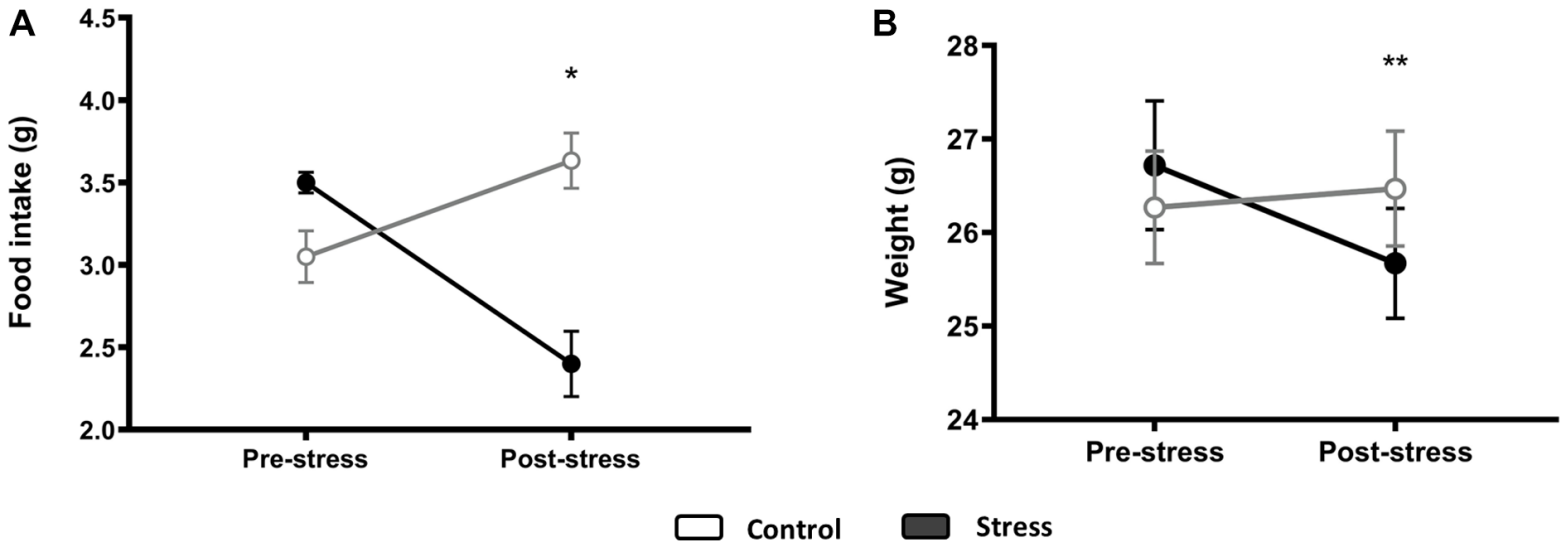
Food intake **(A)** and body weight **(B)** before and after stress. An ANCOVA test was performed, and differences between groups for poststress measurements when controlling for prestress variability are displayed. *p < 0.05, **p < 0.005 (control *N* = 6, stress *N* = 6).

The day after the stress protocol, treated animals showed a decreased food intake compared to the control animals [*F*(1,10) = 10.993, *p* = 0.009, *N* = 12], with no significant effect observed on the prestress food intake [*F*(1,10) = 0.053, *p* = 0.824, *N* = 12] (Figure 5A). As such, body weight was also reduced after stress [*F*(1,10) = 14.516, *p* = 0.004, *N* = 12], and there was a significant difference between pre- and post-stress measurements when controlling for experimental groups [*F*(1,10) = 66.655, *p* < 0.0001, *N* = 12] (Figure 5B).

### 3.2. Cell proliferation and immature neurons were compromised in the hypothalamus after acute stress

**Figure 6:**
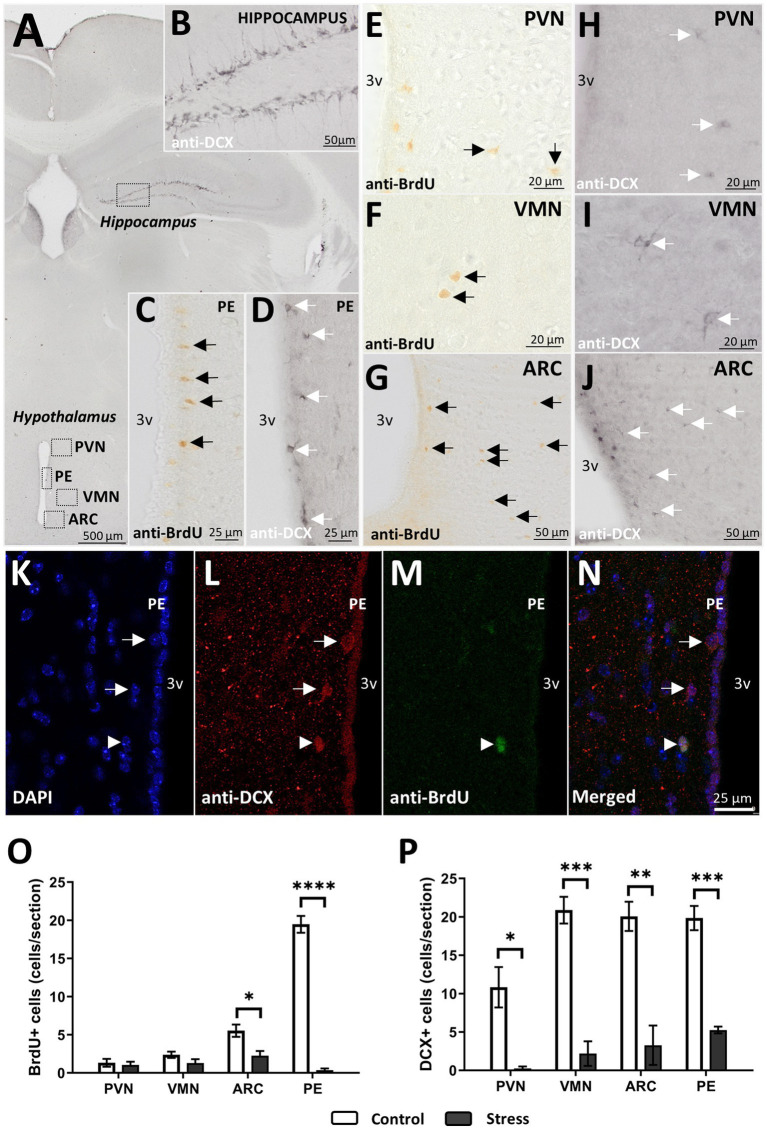
DCX+ cells and proliferation after 24 h. **(A)** Brain map with regions of interest. **(B)** Positive control for immature neurons in the hippocampus. **(C)** Cell proliferation (BrdU+) and **(D)** immature neurons (DCX+) in the periventricular region (PE). **(E–G)** BrdU+ cells and **(H–J)** DCX+ cells in the PVN, VMN, and ARC. **(K–N)** Immunofluorescence staining for DCX+ **(L)** and BrdU+ **(M)** cells in the PE region. Arrows point to DCX+ cells, and triangles point to DCX+ BrdU+ cells in PE. **(O–P)** BrdU+ **(O)** and DCX+ cells after stress treatment in all regions studied. For statistical analysis, *t* test comparisons were made (*p < 0.05, **p < 0.005, ***p < 0.0005, ****p < 0.00005) (control *N* = 4, stress *N* = 4).

Despite cell generation being very low in the area studied (Figures 6E–G) in basal conditions (control group), it became apparent that the generation of new cells (assessed as the number of cells identified 24 h after the last BrdU injection) significantly decreased after stress treatment (Figure 6O). It was not significantly affected in the PVN [*t*(1, 6) = 0.430, *p* = 0.682, *N* = 8] or in the VMN [*t*(1, 6) = 1.647, *p* = 0.150, *N* = 8] (Figure 6O). However, in the ARC, where the number of BrdU+ cells was higher, a significant decrease was observed after stress treatment [*t*(1,6) = 3.239, *p* = 0.017, *N* = 8]. The periventricular region of the hypothalamus (PE) was of interest to find that the majority of BrdU+ cells were arranged just below the ependymal layer and located along the entire wall of the third ventricle (Figure 6C). As in the ARC, the number of new cells was drastically reduced after exposure to stress [*t*(1,6) = 17.087, *p* < 0.001, *N* = 8] (Figure 6O).

To gain more specific insight into hypothalamic neurogenesis, DCX+ cells were studied (Figures 6A,H–J). DCX is a commonly used marker for immature neurons, especially in the hippocampus, where newly generated neurons become more abundant. In control animals, the morphology of DCX+ cells in the hypothalamus differed from its hippocampal counterparts, as the cells were rounder and the ramifications were less apparent (Figures 6H–J vs. Figure 6B). In addition to the aforementioned regions, the periventricular region (PE) was found to be of interest, as most of the young neurons identified as DCX+ were found there (Figure 6D). In addition, it was in the PE where new neurons, identified as BrdU+/DCX+, with a round shape and no processes, were found in control animals (Figures 6K–N).

In any case, the number of hypothalamic immature neurons in the VMN, ARC, and PE was twice as abundant as that in the PVN, with approximately 20 and 10 cells per tissue section, respectively (Figure 6P). After stress treatment, DCX+ cells were considerably reduced in all the regions studied (Figure 6P). More specifically, DCX+ cells were practically abolished in the PVN and reduced to approximately five or fewer cells per section in the VMN, ARC, and PE (Figure 6P).

### 3.3. Arc showed a different microglial profile compared to the PVN and VMN

**Figure 7:**
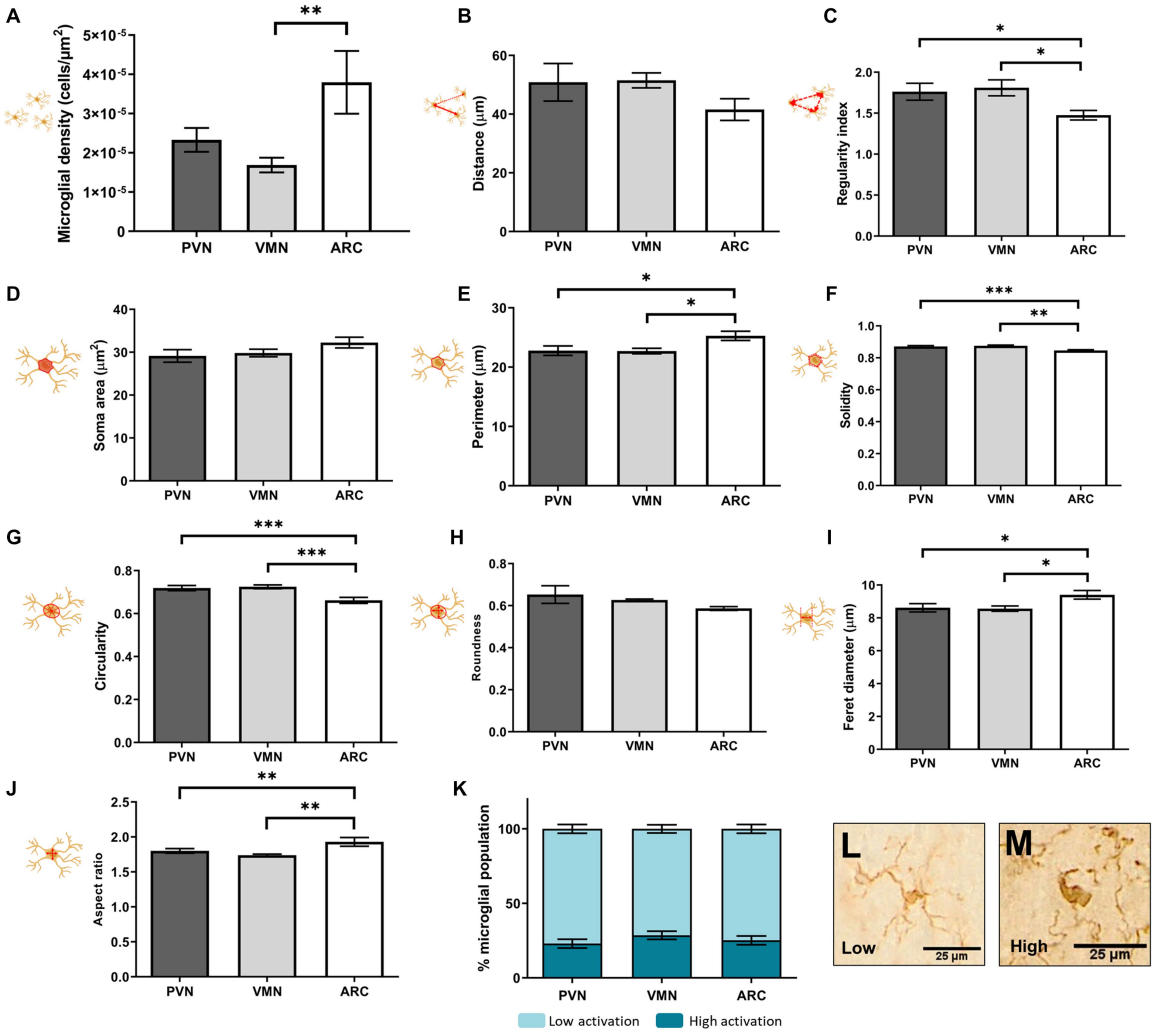
Microglial soma morphological study in control animals: distribution **(A–C)**, size **(D,E)**, and shape **(F–J)**. Percentage of activation as an integrative result for shape and size is shown in **(K)**. **(L–M)** Representative morphologies for low and high clusters in **(K)**. A *t* test was conducted for statistical analysis [*p < 0.05, **p < 0.005, ***p < 0.0005 (*N* = 10)].

The cell morphology study showed differences in cell shape and distribution among the PVN, VMN and ARC in control animals (Figure 7). More specifically, whereas the PVN and VMN showed similar distributions, the ARC showed a higher cell density [*F*(2,27) = 4.550, *p* = 0.019, *N* = 10] (Figure 7A) and a less even distribution compared to the other studied regions (Figure 7C) [*F*(2,27) = 4.144, *p* = 0.027, *n* = 10].

Moreover, differences were found between the ARC and the other two hypothalamic nuclei in cell morphology parameters: cell perimeter [*F*(2,27) = 4.494, *p* = 0.020, *N* = 10] (Figure 7E), solidity [*F*(2,27) = 8.850, *p* = 0.001, *N* = 10] (Figure 7F), circularity [*F*(2,27) = 8.670, *p* = 0.001, *N* = 10] (Figure 7G), Feret diameter [*F*(2,27) = 4.159, *p* = 0.027, *N* = 10] (Figure 7I) and aspect ratio [*F*(2,27) = 5.452, *p* = 0.010, *n* = 10] (Figure 7J). These changes indicated a more irregular cell soma, meaning that the soma had a more ellipsoid shape and a rougher contour in the ARC compared to the PVN and VMN, where cell morphology was found to be similar. However, this was not reflected in the percentage of the microglial population with a highly activated morphology (Figures 7K–M).

### 3.4. Microglia from hypothalamic regions responded differently to acute stress

**Figure 8:**
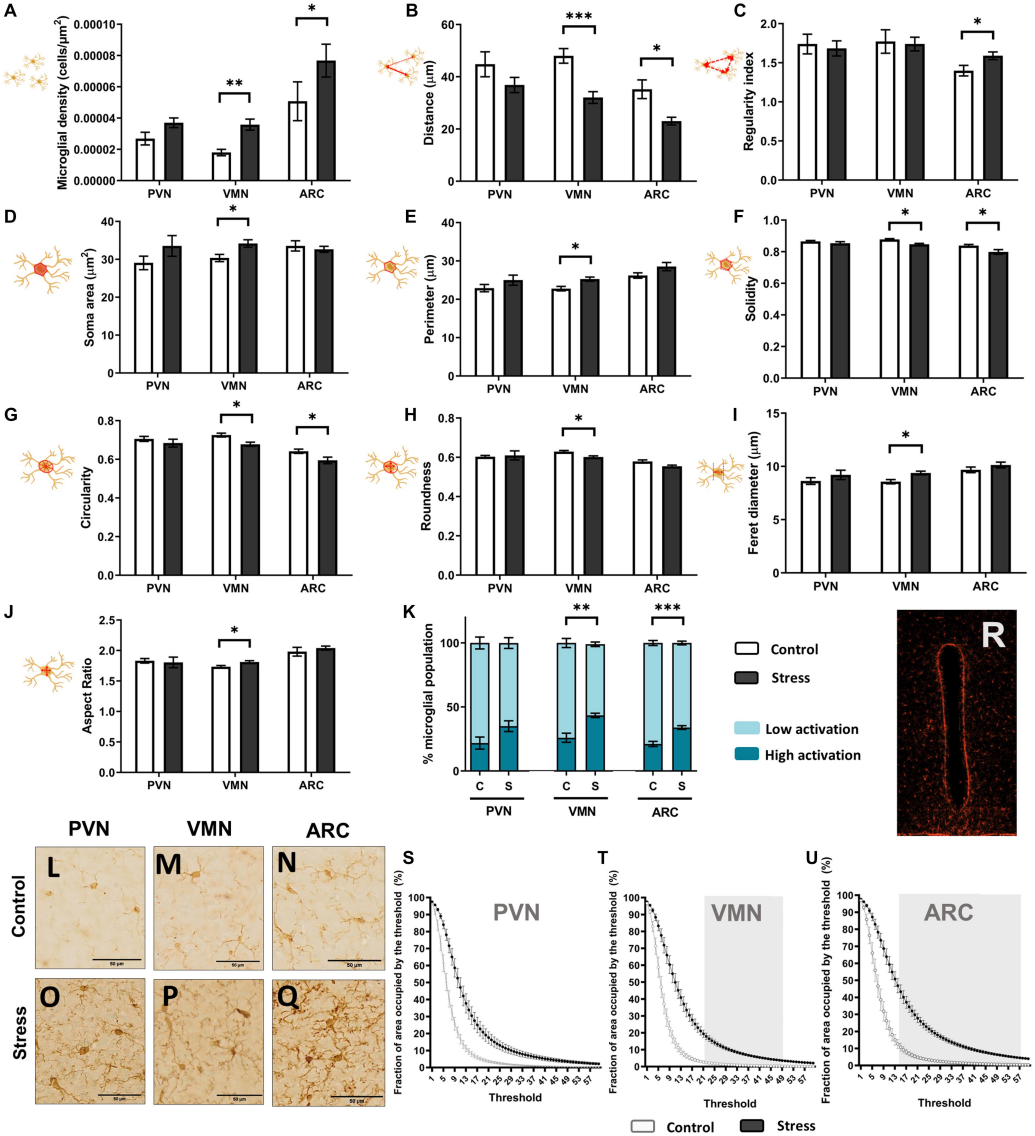
Microglial soma morphological study: distribution **(A–C)**, size **(D,E)**, and shape **(F–J)**. Percentage of activation as an integrative result for shape and size is shown in **(K)**. **(L–Q)** Iba1+ cell comparison between groups in the hypothalamic regions; scale bar 50 μm. A *t* test was conducted for statistical analysis [*p < 0.05, **p < 0.005, ***p < 0.0005 (*N* = 12)]. Otherwise, no significant differences were found. **(R)** Image featuring the third ventricle area occupied by the threshold. Quantitative results for threshold comparisons in the PVN **(S)**, VMN **(T)**, and ARC **(U)**. Significant intervals in the greyscale are shaded in gray, according to a multiple *t* test (per row) using the Holm–Sidak method and *p* < 0.05 (control *N* = 6, stress *N* = 6).

A single acute stressor was sufficient to elicit changes in microglial behavior and distribution, especially in the VMN region but also in ARC (Figure 8). In the PVN, no significant changes were found for any of the studied parameters [*t*(1,10), *p* > 0.05, *N* = 12] (Figure 8). However, the VMN showed significant alterations due to stress in all studied parameters except for the regularity index [*t*(1,10), *p* < 0.05, *N* = 12] (Figure 8). Altogether, this showed an increase in cell density in the region and a shift toward an increase in soma size and a more ameboid phenotype, similar to Figure 7M. This change was evidenced by the increase in cells considered to have a high reactivity phenotype (Figure 8K). Moreover, the rise in the fraction of the area occupied by the threshold aligned with these results (Figure 8T). For the ARC, the cells displayed a denser distribution after stress [*t*(1,10), *p* < 0.05, *N* = 12], and only two of the morphological parameters were altered, namely, solidity [*t*(1,10) = 2.986, *p* = 0.014, *N* = 12] and circularity [*t*(1,10) = 2.295, *p* = 0.044, *N* = 12] (Figures 8F–G). However, the percentage of microglia with a high reactivity phenotype was higher under stress conditions [*t*(1,10) = −5.220, *p* < 0.001, *N* = 12] (Figure 8K), and the fraction of the area occupied by the threshold considerably increased after stress, which was more notable than in the VMN (Figures 8S–U). This indicated that stressed mice had an overall higher level of Iba-1 staining compared to the control. In the VMN, the soma area was increased in stressed mice, which could be partially responsible for the increase in total Iba-1 staining observed after threshold analysis (Figures 8D,T). However, in the ARC, no significant differences were found in the microglial soma area after stress (Figure 8D), although there was an even wider interval for significantly higher total Iba-1 staining (Figure 8U). This finding indicated that microglial processes had grown or thickened due to the stress protocol (Figures 8N,Q). No significant total Iba-1 staining was observed in the PVN (Figure 8S).

Overall, microglia exhibited a morphological response to stress and were observed to be more irregular and denser in the VMN and ARC. Cell processes also appeared to be more apparent in the ARC after stress treatment (Figures 8L–Q).

### 3.5. Reduction in body weight was related to higher microglial density and lower neurogenesis after stress

**Figure 9:**
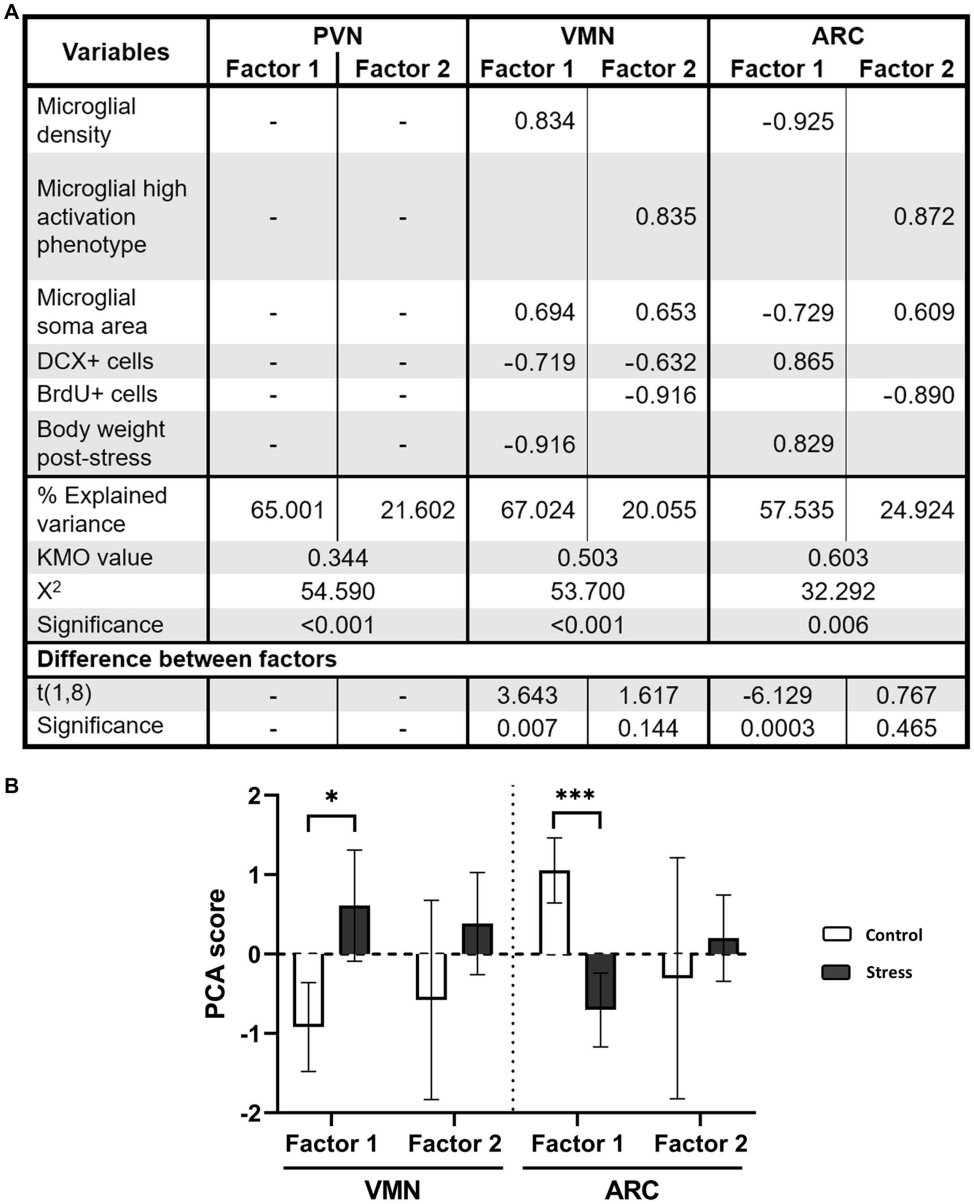
**(A)** PCA for VMN and ARC nuclei. Only factorial scoring with KMO > 0.5 was interpretable; thus, the analysis was not possible for the PVN. Factors were calculated with Varimax rotation with Kaiser normalization. The rotation was converged after 3 iterations for all studied regions. **(B)** PCA scores for each factor and group. For statistical, analysis *t* test comparisons were made [*p < 0.05, ***p < 0.0005 (*N* = 10)].

A PCA revealed that in the PVN, sample adequation criteria were not met (KMO = 0.344), and therefore factors could not be extracted (Figure 9A). However, this was possible in the VMN (KMO = 0.503) and ARC (KMO = 0.603) (Figure 9A). In these regions, two factors were identified, but only the first one was significant between the groups (Figure 9). In the VMN, Factor 1 included all of the variables except for BrdU+ cells and higher microglial reactivity, indicating a relationship among microglial response, neurogenesis and body weight (Figure 9A). According to each variable score, higher microglial reactivity (and especially higher density) was associated with lower neurogenesis and body weight, with the latter two having very low scores. When scores were tested for the different groups, stressed animals displayed a positive result, whereas the score was negative in the control, and this was statistically significant (Figure 9B). This could be read as a higher microglial reactivity and lower neurogenesis and body weight compared to the control. Factor 2 in the VMN pointed toward a relationship between higher microglial reactivity (but not cell density) and lower neurogenesis and especially lower proliferation (Figure 9A). However, differences between experimental groups were not found to be significant (Figure 9A). On the other hand, similar results were found in the ARC. Factor 1 showed that lower microglial density and soma area were related to higher neurogenesis and body weight (Figure 9A). Among the experimental groups, a lower score was found in stressed animals, indicating that a higher microglial cell density and soma area and a lower number of DCX+ cells and body weight were found compared to the control (Figure 9B). Factor 2 described a relationship between a higher proportion of reactive microglia and soma area with lower proliferation in all animals. Nevertheless, no difference was found between experimental groups (Figure 9).

### 3.6. Il-6 was increased in stressed animals after 1 h

According to the high sensitivity molecular analysis performed in brain protein extracts, basal hypothalamic values for the studied chemokines were 5.40 ± 0.36 pg./mL for IL-6, 11.85 ± 0.85 pg./mL for IFN-γ and 5.32 ± 0.36 pg./mL for TNF-α. Thus, the most abundant chemokine under basal conditions was IFN-γ, which showed double the levels of the other inflammatory proteins.

Regarding stress treatment, a one-way ANOVA revealed that there was a statistically significant difference in IL-6 levels between the groups [*F*(3,16) = 10.449, *p* = 0.001, *N* = 20], namely, in the 1 h stress group, which was higher than that of the rest of the groups (LSD, *p* < 0.006). This indicated there was a significant peak in IL-6 1 h after acute stress, which returned to basal levels 24 h after stress (Figure 4B).

Neither IFN-γ [*F*(3,16) = 12.203, *p* = 0.132, *N* = 20] nor TNF-α [*F*(3,16) = 1.270, *p* = 0.322, *N* = 20] showed significant effects in the interactions of Group or Time or in the individual effects of each variable (*p* > 0.05) (Figures 4C,D).

### 3.7. Proteomic changes were enhanced 24 h after stress

Shotgun proteomics revealed that there was already a change in some protein levels after the acute stressor. After 1 h had passed, the animals showed increased expression of three proteins encoded by the genes Anks1b (also known as AIDA-1), Cpne6 and Camkv (Figure 4E). No repression of protein was found in stressed animals meeting the chosen selection criteria. At 24 h after stress, one of the proteins that was overexpressed (Anks1b) was repressed in stressed animals compared to the control (Figure 4F). Other proteins were also repressed, namely, MgII and Hba. Only one protein, STXBP1, was found to be overexpressed in stressed animals (Figure 4F).

After network enrichment, nodes were added to the network by STRING. Topological analysis for these nodes revealed highly connected regions that qualified as clusters, leaving some of the enriched nodes provided by STRING behind. Seeds for each cluster were also identified. Each cluster was given a score based on topology, and the proteins (nodes) were classified into each cluster (Table 1).

**Table 1:** Cluster names, nodes, interactors and scores for topology-based densely connected regions in the enriched network provided by STRING and analysed by MCODE in Cytoscape.

Sample	Cluster name	N. of nodes in cluster	N. of interactors	Cluster score	Genes in cluster
Overexpressed 1 h post-stress	Cluster 1	10	27	6,000	*Mia3, Cdhr4, Brox, Arhgap15, Mon1a, Traip, Apeh, Ip6k1, Hps6, Actl11*
	Cluster 2	21	58	5,800	*Dlg2, Nrxn3, Dlg4, Anks1b, Gpx6, Pacsin1, Shank3, Snrpc, Ank3, Mpp2, Taf11, Gfap, Tcp11, Gad1, Cpne6, Agap2, Scube3, Zfp523, Cntn4, D17Wsu92e, Rbfox3*
	Cluster 3	4	5	3,333	*Mnat1, Arhgap33, Trip4, Tmtc4*
	Cluster 4	3	3	3,000	*Camkv, Mst1r, Rnf123*
	Cluster 5	3	3	3,000	*Syn2, Calm1, Calm3*
Overexpressed 24 h post-stress	Cluster 1	35	581	34,176	*Snap47, Napa, Syt1, Sec22b, Vamp7, Stx6, Stx11, Stx18, Stxbp5, Stx7, Vps33a, Snap29, Vamp1, Stxbp1, Stx1a, Vti1b, Stx3, Stx1b, Vamp3, Cplx2, Vamp2, Snap23, Stx12, Stx5a, Snap25, Stx19, Vamp8, Stx4a, Cplx1, Nsf, Stx16, Stx2*
	Cluster 2	20	146	15,368	*Syn1, Slc18a2, Rab3a, Rims1, Syt4, Ppfia1, Syn3* *Cask, Unc13b, Lin7b, Gad1, Apba1, Gad2, Ppfia3, Lin7a, Lin7c, Slc17a7, Bzrap1, Ppfia2, Ppfia4*
	Cluster 3	14	51	7,846	*Slc32a1, Snap91, Exoc7, Sv2a, Bsn, Grin2b, Syt2* *Unc13a, Syp, Dlg4, Exoc3, Syt5, Slc18a3, Dnm1*
	Cluster 4	6	13	5,200	*Scn2a1, Scn1a, Cdkl5, Pcdh19, Pnpo*
	Cluster 5	4	6	4,000	*Sptan1, Ins1, Ins2, Uso1*
Repressed 24 h post-stress	Cluster 1	31	465	31,000	*Rps26-ps1, Fau, Rps2, Rps13, Rps27a, Rps3a1, Rps11, Rps3, Rps9, Rps6, Hba-a1, Rps5, Rps26, Rps15, Rps17, Rps8, Rps14, Rps12-ps3, Rps20, Rps24, Rps28, Rps29, Rps10, Rps16, Rps25, Rpsa, Rps23, Rps21*
	Cluster 2	19	171	19,000	*Pnliprp1, Lipg, Acsl5, Pnlip, Acsl4, Acsl1, Mgll, Acsbg1, Lipf, Pnpla2, Cel, Pnliprp2, ENSMUSG00000024209, Pnpla3, Acsbg2, Lpl, Lipc, Acsl3*
	Cluster 3	14	91	14,000	*Hbb-bs, Apol9b, Apol7c, Apol7a, Apol9a, Apol11b, Apol7e, Apol10b, Hp, Apol10a, Apol8, Apol11a*
	Cluster 4	11	50	10,000	*Cnr2, Dse, Abhd12, Dagla, Trpv1, Naaa, Napepld, Abhd6, Faah*
	Cluster 5	4	5	3,333	*Hba-a2, Alas2, Hbb-bt, Hbb-y*
	Cluster 6	3	3	3,000	*Mogat1, Agk, Mogat2*
	Cluster 7	3	3	3,000	*Lrp1, Cd163, Hpx*

Nodes refer to protein/gene names.

Based on these results, a functional analysis was obtained for the protein level changes 1 h and 24 h after stress as a more comprehensive and integrative result for the obtained proteins. Proteins that changed 1 h after stress seemed to be only distantly related to each other, as three of the five functional clusters found (Table 1) were deemed insignificant due to low clustering scoring by the algorithm. Thus, only Cluster 2 and Cluster 5 were effectively analysed. They were related to postsynaptic density and calcium-dependent protein binding as the most significant GO terms for the clusters, respectively (Figure 4H).

For the 24 h poststress groups, only overexpression Cluster 4 was found to be insignificant for the enrichment analysis. Functional clusters originating from overexpressed and repressed proteins interacted with each other to form a functional network (Figure 4G). Alterations included an overexpression of proteins related to excitatory synapses and neurotransmitter release and a repression in proteins related to lipid and oxidative metabolism, especially related to endocannabinoid retrograde signaling (Figure 4G).

## 4. Discussion

The hypothalamus is a core structure in regulating the neuroendocrine system and controls key physiological processes such as homeostasis and metabolism (Maggi et al., 2015; Romanov et al., 2020; Bielefeld et al., 2021). In recent years, it has emerged as a new neurogenic area, generating new neurons after development (Pérez-Martín et al., 2010; Lee and Ahima, 2012; Langlet et al., 2013). It is a widely established fact that the hypothalamus responds to environmental stimuli, and a reduction in hypothalamic neurogenesis has been increasingly linked to alterations in hypothalamic functions. However, to our knowledge, the neurogenic response of the hypothalamus to psychological stressors during adulthood has not been studied. The study of acute stress-induced neurological effects during the first phases following exposure to a stressor can provide valuable information to know better the response to stress. For this reason, we have studied the impact of acute and intense stress on hypothalamic neurogenesis and neuroinflammation using an animal model that mimics some of the neurobiological and psychopathological alterations of PTSD (Nieto-Quero et al., unpublished data), an anxiety disorder that has been associated with neurobiological alterations, with the hypothalamus being particularly affected (Mironova et al., 2017). Our data reveal for the first time that even a short-term environmental stimulus such as intense and acute stress (WIRS) could have a significant impact on the hypothalamus by inducing a reduction in proliferation and immature neuronal cells in the parenchymal areas surrounding the 3rd ventricle of the hypothalamus. These changes were accompanied by an increase in microglia reactive to acute stress and a concomitant increase in inflammation, as revealed by the increased in IL-6 levels and the proteomics analysis. These changes were also accompanied by slight changes in the weight and intake of the animals.

Our data revealed that acute stress reduced proliferation in the PE and ARC. Moreover, acute stress reduced the number of DCX+ cells in the parenchyma of all studied areas, indicating a negative effect on hypothalamic neurogenesis. The hypothalamus is a core structure in the regulation of the neuroendocrine system and controls key physiological processes such as homeostasis and metabolism (Maggi et al., 2015; Romanov et al., 2020; Bielefeld et al., 2021). Consequently, stimuli that alter neurogenesis may have functional effects on hypothalamic functioning. Importantly, it has been shown that environmental manipulation that reduced hypothalamic neurogenesis induced changes in leptin signaling, body adiposity, and diet responsivity (Maniam et al., 2016; Yam et al., 2017). Direct inhibition of adult PE neurogenesis results in impaired hypothalamic functions, resulting in problems in weight regulation, energy metabolism and body adiposity and alterations in the response to a high-fat diet (Lee and Ahima, 2012; McNay et al., 2012; Yoo et al., 2020). Inhibition of cell proliferation in the ventral regions of the hypothalamus caused weight loss and food intake deficits in mice. These effects revealed that hypothalamic newborn/immature neurons may play a critical role in the regulation of energy balance and be specifically required for weight management (McNay et al., 2012). Therefore, when hypothalamic plasticity is reduced, hypothalamic network adaptability may become compromised, resulting in simultaneous defective energy balance. It is therefore not surprising that acute stress can lead to homeostatic changes. Indeed, acute stress had a slight effect on intake and body weight, indicating that it had an impact on metabolism. Thus, despite very low levels of hypothalamic neurogenesis under basal conditions, small changes in this process may have potentially outsized effects on physiology and behavior. However, more long-term studies are needed to understand the extent of these effects and the long-term impact of intense acute stress on hypothalamic plasticity.

However, how stress impacts hypothalamic neurogenesis remains unknown. Recent studies, which focus on other neurogenic regions, indicate that changes in inflammation, mediated primarily via microglial cells, may play an essential role in this process (Sierra et al., 2010). Microglia are myeloid cells that may rapidly respond to even minor changes in the microenvironment. Among their numerous functions, microglia play a role in regulating adult neurogenesis (Ekdahl et al., 2003; Kempermann and Neumann, 2003; Sierra et al., 2010; Valero et al., 2016; Diaz-Aparicio et al., 2020). In both the hypothalamus and other neurogenic regions, in response to stress, microglia undergo several morphological changes (Sugama et al., 2007; Walker et al., 2013), which, with exceptions, have been linked to functional changes. Here, we characterized the microglial cell population in the PVN, VMN and ARC. Under basal conditions, the degree of microglial activation is very similar among the three nuclei studied, although, as expected, the number and morphology of microglia in the ARC nucleus indicate a slightly higher degree of activation. The ARC is a circumventricular organ strategically placed to detect even small signals circulating in the blood and report on peripheral metabolic status (Djogo et al., 2016; García-Cáceres et al., 2019). After acute stress, PVN microglia were surprisingly unresponsive to the stressor, despite their role in regulating the release of trophic hormones in response to several inputs from both the CNS and peripheral system (Sawchenko and Swanson, 1982; Biag et al., 2012; Bouyer and Simerly, 2013; Rosin and Kurrasch, 2019). In contrast, microglia in the VMN and in the ARC responded to the acute stress protocol. As such, stress induced an increase in microglial density and a reduction in the distances between microglia in both hypothalamic nuclei. In parallel, a change in microglial morphology compatible with an amoeboid appearance was observed, which together with the changes in total microglial staining suggests an increase in WIRS-reactive microglia. This assumption was corroborated by clustering analyses, which indicated an increased proportion of stress-reactive microglia. However, the use of markers of activated microglia by immunohistochemistry could be a good complement to the morphological studies, allowing better characterization of the microglia in response to stress.

Moreover, cytokine determination revealed a significant increase in the level of IL-6 1 h after stress. In this sense, although IL-6 may act as both proinflammatory and anti-inflammatory molecule, acute stress is an “injury-like stimuli,” under which IL-6 can act as a pro-inflammatory cytokine (Bobbo et al., 2019). Marked microglial activation together with a concomitant increase in IL-6 levels indicate that WIRS induced an inflammatory response in the VMN and the ARC.

Microglia may act as injury-responsive mediators of hypothalamic dysfunction. Environmental factors modulate microglia, which can regulate brain function, namely, neurogenesis, which participates in different stages and is necessary for proper adjustment of the process (Gemma and Bachstetter, 2013; Valero et al., 2016; de Miranda et al., 2017; Frank et al., 2019; Rosin and Kurrasch, 2019; Diaz-Aparicio et al., 2020). Although there are some differences, we have shown that in both the VMN and ARC, higher microglial density and larger area are associated with a lower number of immature neurons and lower body weight on a regular basis. In addition, there were statistically significant differences between controls and stressed animals in Factor 1, indicating that stressed animals had a higher number of WIRS-reactive microglia, a decreased number of DCX+ cells and a lower body weight.

On the other hand, we tried to determine whether proteomic changes take place at 1 h and 24 h after the application of WIRS. The data revealed that WIRS induced changes in the hypothalamic proteome, modifying the abundance of three and four proteins after 1 h or 24 h of stress application, respectively.

The modified protein data revealed that 1 h after stress, the levels of proteins were increased, including Cpne6, Camkv and AIDA-1 (Anks1b). These proteins have been directly or indirectly linked to calcium regulation, neuroplasticity and transcriptional activity (Liang et al., 2016; Barylko et al., 2022). More specifically, Cpne6 is a calcium sensor implicated in long-term potentiation and dendritic spine morphology in the hippocampus, and its deficiency has been linked to memory deficits and plasticity (Reinhard et al., 2016; Burk et al., 2018). Camkv has been found in mRNA form in vesicles near dendritic spines and is activated under neuronal activation to further activate gene expression via the Rho transcription factor in the hippocampus. Its deficiency has been linked to memory deficits and synaptic impairment (Liang et al., 2016). AIDA-1, has been associated with the postsynaptic density scaffold, which is key for new synapse formation or already existent reinforcement. It also regulates NMDA receptor subunit composition, promoting a greater GluN2A presence and transport of GluN2B to the endoplasmic reticulum in the hippocampus (Younis et al., 2019). GluN2B subunit transport to the ER could be related to upregulation in ER transport found in the enrichment analysis 24 h after stress. This composition has been found to be more prevalent in mature neurons than in young neurons (Sun et al., 2018), which could indicate maturation in the neurogenic process of stressed animals. Among the impairments that have been associated with high levels of these proteins is the development of inflammation by an increase in IL-6 (Ahola-Olli et al., 2017) and an impairment in body weight control (Younis et al., 2019). The normalization of Cpne6 and Camkv levels and downregulation of AIDA-1 expression observed 24 h after stress coincided temporally with the return to baseline values in IL-6 and corroborated this effect. These individual findings in the literature seem to be supported by the enrichment analysis, which noted the activation of synaptic density-related proteins as well as calcium-dependent protein binding. This process is highly relevant for synapse homeostasis, as well as for synaptic generation and plasticity (Ho et al., 2000; Borger et al., 2014).

On the other hand, among the proteins under- or overexpressed 24 h after the application of the stressor, the Hba protein, a subunit of hemoglobin, is noteworthy. Expression of this protein in various brain regions (Genecards, HBA1) has been linked to the regulation of oxidative stress and has been identified in various neurodegenerative diseases (Zheng et al., 2022). The functional network indicated that haem group capture from plasma as well as oxygen transportation and haem group binding were downregulated in stressed mice. This could be indicative of poor oxygen conditions in the hypothalamus similar to ischaemia, which triggers necrotic cell death and a subsequent inflammatory response, including reactive oxygen species and inflammatory cytokine production (Kawabori and Yenari, 2015). This hostile environment could lead to poor neurogenic development and support lower immature neuron findings. Moreover, 24 h after stressor application, a reduction in Mgll, a lipase that has been associated with inactivation of endocannabinoids, has been observed (Dinh et al., 2002). Endocannabinoids have been associated with the control of intake, appetite, and energy balance in the hypothalamus. Enrichment analysis further supported this finding, pointing toward a reduction in lipid-transporting proteins and lipid degradation, as well as in the endocannabinoid system. Lipid metabolism in the brain is tightly connected to brain injury and inflammation (Adibhatla and Hatcher, 2008), and alterations in lipid availability and transportation (for example, via APOE proteins) have been found to increase inflammatory disorders such as Alzheimer’s (Zhong et al., 2019; Duro et al., 2022). This could explain, at least in part, the reduction in weight and intake observed in stressed animals. Moreover, an upregulation of neurotransmitter release and the active zone in excitatory synapses was found 24 h after stress, which together with the upregulation in synaptic density and calcium-dependent protein binding found 1 h after stress, could indicate prolonged activation in excitatory synapses. Although excitation is related to long-term potentiation processes (Pradier et al., 2018), in this study, we found evidence supporting NMDA but not AMPA regulation, suggesting other possible mechanisms. One of them could be excitotoxicity, where an excess in neurotransmitter activity can lead to cell death and hypoxia (Choi, 2020), which we found in our results. Moreover, excitotoxicity has been linked to the CB1 endocannabinoid receptor and neurogenic progenitor proliferation (Aguado et al., 2007), which adds to the evidence found in this study. However, given the short time elapsed between the application of the stressor and the measurements taken, further studies are needed to determine the effect of an acute and intense stressor on metabolism. In this regard, the changes observed in microglia, together with the increase in inflammatory cytokines and proteomic changes could indicate an increase in the inflammatory response following WIRS application for 2 h.

In conclusion, our data revealed that following an acute stressor, particularly an intense stressor that may be considered to establish an animal model of PTSD, many changes that provide valuable insights into the mechanisms involved in the impact of the stress response on the physiologically relevant hypothalamus were initiated (Campbell et al., 2017). Thus, our results show that stress exposure restricted to 2 h induced structural (morphological changes in microglia), functional (protein changes and plasticity), and metabolic consequences (reduction of intake and body weight). These acute stress-related changes may contribute to explaining how limited exposure to acute stress results in the metabolic and autonomic impairment associated with stress-related disorders. Furthermore, we have shown, at least in the VMN and ARC, that the microglial response is related to stress-induced neurogenic changes and metabolic impairment. Taken together, these data reveal that even a short-term stressor can have a significant impact on the hypothalamus, a central regulator of the neuroendocrine system that controls key physiological processes and may shed light on the neurobiological mechanisms involved in the development of PTSD.

## Data availability statement

The data presented in this study are deposited and made publicly available in: Dataset Orbitrap Raw-Data Stress Hypothalamus(1h-24h): https://dx.doi.org/10.24310/riuma.26229.

## Ethics statement

The animal study was reviewed and approved by University of Malaga Ethical Committe (CEUMA 51-2018-A) and corroborated by the Agriculture, Farming, Fishing and Sustainable Development Council (10/06/2019/114).

## Author contributions

MII-L, AN-Q, and EZ-I: procedures with animals. MII-L, AN-Q, and PC-P: immuno assays and cell counting. AN-Q and MII-L: hippocampal molecules analysis. MC, JM, CP, and MP-M: design and supervision of the experiments, and draft of the article. MII-L, AN-Q, CP, and MP-M: writing of the article. CP and MP-M: funding for the study. All authors contributed to the article and approved the submitted version.

## Funding

This study was supported by Consejería de Conocimiento, Investigación y Universidades, Junta de Andalucía P20_00460-Co-financing by the European Regional Development Fund (ERDF/FEDER) to CP; Ministerio de Ciencia e Innovación—Plan Nacional I+D+I from Spain Grant PID2020-117464RB-I00 funded by MCIN/AEI/10.13039/501100011033 to CP and MP-M; FEDER/Junta de Andalucía—Proyectos I+D+I en el marco del Programa Operativo FEDER Andalucía 2014-2020 (UMA20-FEDERJA-112) to CP and MP-M.

## Conflict of interest

The authors declare that the research was conducted in the absence of any commercial or financial relationships that could be construed as a potential conflict of interest.

## Publisher’s note

All claims expressed in this article are solely those of the authors and do not necessarily represent those of their affiliated organizations, or those of the publisher, the editors and the reviewers. Any product that may be evaluated in this article, or claim that may be made by its manufacturer, is not guaranteed or endorsed by the publisher.
