# Corrigendum: New insights into hypothalamic neurogenesis disruption after acute and intense stress: implications for microglia and inflammation

**DOI:** 10.3389/fnins.2023.1335034

**Published:** 2023-11-23

**Authors:** María Inmaculada Infantes-López, Andrea Nieto-Quero, Patricia Chaves-Peña, Emma Zambrana-Infantes, Manuel Cifuentes, Javier Márquez, Carmen Pedraza, Margarita Pérez-Martín

**Affiliations:** ^1^Departamento de Biología Celular, Genética y Fisiología, Universidad de Málaga, Málaga, Spain; ^2^Instituto de Investigación Biomédica de Málaga y Plataforma en Nanomedicina–IBIMA Plataforma Bionand, Málaga, Spain; ^3^Departamento de Psicobiología y Metodología de las Ciencias del Comportamiento, Universidad de Málaga, Málaga, Spain; ^4^Departamento de Biología Molecular y Bioquímica, Canceromics Lab, Universidad de Málaga, Málaga, Spain

**Keywords:** neurogenesis, microglia, inflammation, acute stress, hypothalamus, hypothalamic proteomic profile

In the published article, there was an error in the Funding statement. This is an error both in the reference to the last project and in the order, which should be the first to be cited and not the last. “This study was supported by Ministerio de Ciencia e Innovación—Plan Nacional I+D+I from Spain Grant PID2020-117464RB-I00 funded by MCIN/AEI/10.13039/501100011033 and, as appropriate, by “ERDF A way of making Europe”, by the “European Union” or by the “European Union NextGenerationEU/PRTR” to CP and MP-M; FEDER/Junta de Andalucía—Proyectos I+D+I en el marco del Programa Operativo FEDER Andalucía 2014–2020 (UMA20-FEDERJA-112) to CP and MP-M; and Consejería de Conocimiento, Investigación y Universidades, Junta de Andalucía (P20_00460) to CP.” The correct Funding statement appears below.

